# Retraction Note: Hsa-miR-520d induces hepatoma cells to form normal liver tissues via a stemness-mediated process

**DOI:** 10.1038/s41598-018-32380-8

**Published:** 2018-10-09

**Authors:** Satoshi Tsuno, Xinhui Wang, Kohei Shomori, Junichi Hasegawa, Norimasa Miura

**Affiliations:** 1 Division of Pharmacotherapeutics, Department of Pathophysiological & Therapeutic Science, 86 Nishicho, Yonago, Tottori 683-8503 Japan; 2 Division of Organ Pathology, Tottori University, 86 Nishicho, Yonago, Tottori 683-8503 Japan

Retraction of: *Scientific Reports* 10.1038/srep03852, published online 24 January 2014

This Article is being retracted by the Editors. An investigation at the Tottori University^1^ established that Figures 1C, S2H and S2H contain duplicate images of Western blotting data corresponding to different experimental conditions, as well as inappropriate manipulation of these images. The Authors are unable to present original raw data, and therefore the conclusions of the paper cannot be considered reliable.

The Authors do not agree with the retraction.
